# Prevention of postpartum haemorrhage by community-based auxiliary midwives in hard-to-reach areas of Myanmar: a qualitative inquiry into acceptability and feasibility of task shifting

**DOI:** 10.1186/s12884-017-1324-6

**Published:** 2017-05-17

**Authors:** Kyu Kyu Than, Yasmin Mohamed, Victoria Oliver, Theingi Myint, Thazin La, James G. Beeson, Stanley Luchters

**Affiliations:** 1 Burnet Institute, Melbourne, Australia; 2 Department of Medicine, University of Melbourne, Melbourne, Australia; 3 Monash Institute of Pharmaceutical Sciences, Monash University, Melbourne, Australia; 4 Department of Public Health, Ministry of Health and Sports, Nay Pyi Taw, Myanmar; 5 Department of Epidemiology and Preventive Medicine and Central Clinical School, Monash University, Melbourne, Australia; 6 International Centre for Reproductive Health, Department of Uro-Gynaecology, Ghent University, Ghent, Belgium

**Keywords:** Maternal health, Community-based care, Oral misoprostol, Postpartum haemorrhage, Auxiliary midwives, Task shifting, Qualitative inquiry, Myanmar

## Abstract

**Background:**

In Myanmar, postpartum haemorrhage is the leading cause of maternal mortality and contributes to around 30% of all maternal deaths. The World Health Organization recommends training and supporting auxiliary midwives to administer oral misoprostol for prevention of postpartum haemorrhage in resource-limited settings. However, use of misoprostol by auxiliary midwives has not formally been approved in Myanmar. Our study aimed to explore community and provider perspectives on the roles of auxiliary midwives and community-level provision of oral misoprostol by auxiliary midwives.

**Methods:**

A qualitative inquiry was conducted in Ngape Township, Myanmar. A total of 15 focus group discussions with midwives, auxiliary midwives, community members and mothers with children under the age of three were conducted. Ten key informant interviews were performed with national, district and township level health planners and implementers of maternal and child health services. All audio recordings were transcribed verbatim in Myanmar language. Transcripts of focus group discussions were fully translated into English before coding, while key informants’ data were coded in Myanmar language. Thematic analysis was done using ATLAS.ti software.

**Results:**

Home births are common and auxiliary midwives were perceived as an essential care provider during childbirth in hard-to-reach areas. Main reasons provided were that auxiliary midwives are more accessible than midwives, live in the hard-to-reach areas, and are integrated in the community and well connected with midwives. Auxiliary midwives generally reported that their training involved instruction on active management of the third stage of labour, including use of misoprostol, but not all auxiliary midwives reported using misoprostol in practice. Supportive reasons for task-shifting administration of oral misoprostol to auxiliary midwives included discussions around the good relationship and trust between auxiliary midwives and midwives, whereby midwives felt confident distributing misoprostol to auxiliary midwives. However, the lack of clear government-level written permission to distribute the drug was perceived as a barrier to task shifting.

**Conclusion:**

This study highlights the acceptability of misoprostol use by auxiliary midwives to prevent postpartum haemorrhage, and findings suggest that it should be considered as a promising intervention for task shifting in Myanmar.

**Electronic supplementary material:**

The online version of this article (doi:10.1186/s12884-017-1324-6) contains supplementary material, which is available to authorized users.

## Background

Maternal mortality remains a major challenge to health systems worldwide. Globally, there were an estimated 303,000 maternal deaths in 2015 [1]. The leading cause of maternal mortality in low-income countries is postpartum haemorrhage, which is the primary cause of nearly one fifth of all maternal deaths worldwide [2]. Postpartum haemorrhage is commonly defined as a blood loss of 500 ml or more within 24 h after birth. In many cases, postpartum haemorrhage after birth is preventable through use of prophylactic uterotonics during the third stage of labour with timely and appropriate care and management [3].

In Myanmar, it is estimated that the maternal mortality ratio decreased from 360 to 200 per 100,000 live births between 2000 and 2013 [1]. However, according to a nationwide cause-specific maternal mortality survey, postpartum haemorrhage remains responsible for an estimated 30% of all maternal deaths, making it the leading cause of maternal mortality in the country [4]. Urgent actions are being taken by the Government of Myanmar to bring down the high rate of maternal mortality, recognising the human resource shortage in hard-to-reach areas as one of the main causes. Inadequate health literacy in the community, being responsible for too many women, and poor accessibility are some of the challenges faced by skilled birth attendants assigned to rural hard-to-reach areas [5]. One of the mechanisms that the Ministry of Health has employed is to promote the existence of auxiliary midwives (AMWs) with the aim of “one AMW to be trained in every village” by 2016 [6]. AMWs are categorized as unpaid volunteer health workers and have been part of the health system since the late 1970s [7]. AMWs are mostly local women with secondary level of education, who have been selected and trained under the guidance of the Township Health Department for three months in theory and three months in practical skills related to antenatal care, uncomplicated deliveries and postnatal care. The main role of AMWs is to perform preventive care such as counselling of mothers on safe motherhood and promoting delivery with a skilled attendant. AMWs are to assist midwives (MW), who are skilled birth attendants from the formal health sector.

When a skilled birth attendant is available, the most effective intervention for preventing postpartum haemorrhage is active management of third stage of labour which includes administration of a uterotonic drug after the birth of the baby, controlled cord traction, and uterine massage. Active management of third stage of labour using oxytocin injection as the preferred uterotonic is current best practice for prevention of postpartum haemorrhage [3, 8, 9]. However, the need for cold chain storage and a skilled provider to administer an injection hinders the distribution and usage of oxytocin in many resource-limited settings, particularly in more remote communities [10].

Misoprostol is an oral uterotonic drug suggested as a substitute to oxytocin in low resource settings [11, 12]. As this drug does not require cold storage and can be administered orally, it has been used and proven to be effective when administered by community health workers to prevent postpartum haemorrhage in several countries [13–16]. Current World Health Organization (WHO) task shifting guidelines specifically recommend misoprostol use by AMWs in situations where there are no skilled birth attendants and home births are common [17]. In Myanmar, 70% of the population resides in rural areas where home births are a common practice [18]. Although AMWs in Myanmar are categorized as unskilled birth attendants, current training manuals include the topic on active management of the third stage of labour with the use of misoprostol [19]. However, use of uterotonics including misoprostol by AMWs remains controversial due to concerns regarding the management of possible side-effects such as fever and chills, and the potential use of misoprostol to induce abortion. This study aimed to explore community and provider perspectives on the role of AMWs and explore community-level provision of oral misoprostol by AMWs.

## Methods

To understand the barriers and facilitators towards task shifting of AMWs in Myanmar, we undertook a descriptive mixed methods study. This paper reports on the qualitative research component of the study.

The qualitative inquiry was conducted in Ngape Township (an administrative subdivision of a district) in Myanmar. Ngape Township was purposefully chosen because it is a hard-to-reach township, some 16 h driving from Yangon. Ngape Township has an estimated population of 46,572 and has one township hospital, one station hospital and three rural health centres.

The consolidated criteria for reporting qualitative research (COREQ) checklist was used to report the methodology and findings of the study [20].

The research team consisted of one experienced qualitative researcher who moderated all interviews and focus groups, and four research assistants involved in note-taking and transcribing the audiotapes. Apart from one research assistant, all were female. Two research assistants were medical doctors and two were non-medical, all with some level of experience in conducting in-depth interviews (IDIs) and focus group discussions (FGDs). The research team participated in a three-day refresher training on the background and rationale of the study, its objectives, ethical considerations and on strengthening certain qualitative research techniques more specifically.

The topics that were covered in the interviews and focus groups were beliefs and practices around childbirth, community preference of birth care provider, birth place, role of provider in antenatal, childbirth and postpartum care, beliefs and practices around postpartum haemorrhage and other complications, relationship with health care providers, perspectives towards AMWs, and provider and community perspectives towards task shifting of misoprostol to AMWs. Final interview guides are available at Additional file 1. Pre-testing of the interview and focus group guides was done in Thanlyin Township (a semi-rural area in Yangon Division) to identify the nature of the interview/focus group and the content of the interview or focus group guide. From the pre-test, it was learnt that conducting FGDs with the AMWs in the hospital setting was not conducive to the discussions as they were reluctant to talk about issues related to relationships with health care providers and actual practices in the community. Therefore, apart from the two FGDs with the MWs, which were done in the township hospital, all the other FGDs were done in community gathering places of the respective villages and the office of an international NGO in Ngape Township. All the individual interviews were done in private places chosen by the interviewee, and were mostly carried out in the offices of interviewees.

Five advocacy meetings were undertaken with the district and township level authorities before data were collected. A total of ten key informants (three national level health planners, five district and township level health planners and implementers, and two from the Three Millennium Development Goal (3MDG) fund who were involved in maternal and child health program implementation) were conducted. Moreover, 15 FGDs (two with MWs, five with AMWs, four with community members and four with mothers of children under the age of three years) were done from both hard-to-reach and non-hard-to-reach areas as the main dimension for sampling. Each FGD consisted of between five and twelve participants. The criteria for categorization of the hard-to-reach and non-hard-to-reach villages are based on the geographical hard-to-reach definition used by the 3MDG programme based on scores allocated for travelling time to the nearest facility, mode of travel, transport charges and roads affected by seasonal variation. A total of 12 points are given and villages scoring 0 to 3 are considered as non-hard-to-reach, while 4 and above as hard-to-reach villages [21].

As all the research team members were Myanmar nationals, there were no language or cultural barriers. The process of the research team members explaining ethical procedures and permissions granted to the participants during the first session created reluctance among many participants in the beginning of the interviews and focus groups, as they saw the interviewers as academic professionals, creating a power imbalance. This was mitigated by first talking to them about their personal backgrounds and demonstrating an interest in their views regarding the topic, which took around five to ten minutes. It is still acknowledged that responses from participants may have been influenced by their perception of the research team.

All the FGDs were audio recorded with written informed consent from the participants. Three interviewees refused to be audio recorded; all other IDIs were audio recorded. Note taking was done for all the interviews and FGDs. All audio recordings were transcribed verbatim in Myanmar language from the digital recorders by the note takers and checked against field notes for consistency. The durations of the FGDs and IDIs ranged from 30 min to 90 min with an average duration of 50 min. Data were collected until it was felt that data saturation was obtained. During the data collection process, daily discussions were made around the main themes using a matrix in Myanmar language. These discussions afforded the opportunity to undertake preliminary interpretation of the data collected while it was still fresh in the minds of the research staff. Before the actual coding, all transcripts were read and reread by the principal author in Myanmar language and all the translated versions of the transcripts were read and reread by the other coders. Transcripts of FGDs were fully translated into English before coding, while remaining data were coded in Myanmar language using ATLAS.ti software. Two data coders coded the transcripts. Reliability coding was set at 80% agreement and the inter-coder reliability was found to be over 80%. This approach balanced the differing views of the researchers in the study. During the time of coding, any unclear information regarding the transcripts and context was verified and clarified with the primary author who has knowledge of the context. The primary coding structure was developed around conceptual codes and sub-codes identifying the key concepts and essential dimensions of the main topic domains. In some of the topics the sub-code level went down to three levels under the main topic. Relationship codes were also found identifying links between other concepts coded with the conceptual codes. Participant characteristics (key informants, MWs, AMWs, mothers and community members) were also considered during the coding process. Before finalising the code structure, the two researchers who coded the transcripts collaboratively reviewed the coding structure and agreed on the final version. Main themes were pre-identified using the focus group discussion and the individual interview guides and emerging themes were also noted and discussed. Quotations are used to support the study findings and to enhance understanding of the local context.

## Results

### Study participants

Data were collected over a 7-month period between July 2015 and February 2016. Ten participating key informants came from the national Department of Health, and from district and township level health departments involved in maternal and child health planning and implementation (Table 1). District and regional level health departments provide supervisory and technical support to the township level and guide the process of AMW recruitment, supervision, training and decision making towards AMW roles and tasks. Township level health departments mainly manage the township health system which is the backbone of primary health care, provide comprehensive health services at the local level and is predominantly responsible for management of the AMW activities [22]. Key informants had between five and 37 years of experience in the health service.

**Table 1:** Overview of study participants of the focus group discussions and in-depth interviews

Category	Number of Focus Group Discussions	Total no of participants	Age range of participants
Auxiliary Midwives (AMWs)	5	33	19–52 years
Midwives (MWs)	2	15	24–55 years
Mothers with children under three years of age	4	29	21–37 years
Community members	4	36	21–60 years
Key informants	–	10	31–67 years

Fifteen MWs and 33 AMWs participated in two and five FGDs respectively. Thirty-six community members participated in the FGDs, 18 being male and 18 female. Community members comprised of local people who are knowledgeable about their village such as community leaders, teachers, village health committee members and elders.

### Community attitudes and practices towards childbirth

Our inquiry identified that many women in the study township preferred a home birth. The main reasons for choosing a home birth were family and community support given at the time of childbirth. In hard-to-reach villages, a village was still perceived as one large family with social and traditional closeness to one another. It was perceived that during childbirth, women who may or may not be a relative are always there to lend a helping hand. Participants in the study mentioned that giving birth surrounded by relatives and neighbours provided them with strength and courage.

*“ ….when we have labour pain we call: “Mom, sisters, please come. I have pain.” We also call the neighbours. I feel strong and they cheer me and help me shout”*

(a woman with four children in FGD with mothers)

Another reason given in favour of home births was that there is no one to look after the children or household if mothers would leave the house. For a facility delivery, there tended to be a need for an accompanying person to look after the woman and at the same time someone to look after her children at home. Someone to stay with the children or take care of the household during the facility delivery was hard to find, while for home deliveries women can easily come and attend the delivery as they live near to one another and can come when they have free time.

*“…they prefer home deliveries…..most of the people live in the farms and not in the village…..who will look after their live stocks like pigs and chickens…who will look after their children?”*

(AMW from non hard-to-reach village during FGD with AMWs)

Community members and providers in hard-to-reach areas mentioned long distances and travelling time to reach the nearest hospital as a barrier to facility births. In these areas, traveling to the nearest hospital could take over 6–10 h of walking before reaching a road where transport needs to be arranged. Women in these hard-to-reach areas were described by MWs as the hardest community groups to send to hospital when requiring emergency care. Planned facility based deliveries for high-risk women are recommended by the AMWs and MWs. It was reported that many of the women who lived near a facility were more willing to go for a facility delivery, whereas women in hard-to-reach areas only go to a health facility for complications and emergencies.

*“I got labour pain and waited for about 1 day and 1 night hoping to deliver the baby at home. The TBA [traditional birth attendant] didn’t know what was happening. When examined by the AMW and didn’t see the head of the baby as well. I was told I must go to the hospital… So, I hired the car and went to the hospital”*

(a woman who had facility delivery from hard-to-reach area in the FGD with mothers)

It was not only due to distance, but also due to the costs associated with transport and hospitalization. Women reported that the only reason to give birth at a facility was in situations of a life-threatening emergency.

*“I had to go to the hospital because my placenta did not come out after the baby was born… I was scared but the AMW said we must go… or it will cause my life”*

(a woman who had complications during birth in the FGD with mothers)

### Care providers at home births

The main providers attending home births in the study area are AMWs, MWs and traditional birth attendants. Few reported on the use of traditional birth attendants to manage the home deliveries. Currently, traditional birth attendants are reported to be less frequently used for assisting with deliveries, but considered as helpers for the daily cooking and cleaning activities for some of the wealthier families in the village. In villages where the MW is present, home births are mainly attended by the MWs. However, in villages where there is no MW, and particularly in hard-to-reach areas, the main health care providers for home births are AMWs. In some of the villages where there are both MWs and AMWs, the AMWs often assist the MWs during delivery.

*“mostly the mothers call the AMWs first because they live in the village but the AMWs under my supervision always inform me with a phone and I also attend the delivery together.”*

(MW from a hard-to-reach area during FGD with MWs)

### Perspectives towards AMWs

According to the mothers and the other community members, AMWs were easily accessible as they lived within the community. They described AMWs as ‘natives who live close to them’ and who can be called upon 24 h a day, seven days a week. Having a village-owned AMW was considered an asset to the village. Mothers in the community also mentioned that AMWs are trustworthy and skilful members of the community. AMWs are well-recognised by the community for their care during the time of need not only for pregnant women but for the village as a whole.

*“She [AMW] lived in this village all her life. She delivered all three of my babies and she is very skilful. Also, she [AMW] has very good relationship with [midwife] and if needed she also calls her and attend the deliveries together.”*

(Female community member during FGD with community members)

Acceptability of AMWs was generally high among key informants from all levels of the health system. MWs and other township level health care providers mentioned that AMWs are promising health care providers for the community, especially in the more remote areas where there is no MW. MWs reported relying on the AMWs for all services rendered to the mother and the child and MWs appreciated their assistance.

*“AMWs are essential especially in places where we can’t go. Mothers may want to deliver with us… but in reality it is very far and we cannot be there, so they usually deliver with the AMWs. If they need help, they send someone (flag person) to call us for help…. when it comes to delivery, they [women] can’t wait and have no time to call us in advance.”*

(MW assigned to a hard-to-reach area during FGD with MWs)

Community members and AMWs in the study mentioned that in some of the villages, MWs were only able to come once a month for immunization. Reluctance of MWs to stay in villages, and barriers to access resulting from long distances and tough travelling circumstances were major factors.

*“Our village is quite far, although a midwife is assigned, she rarely comes, she only comes for immunization… not only her… none of the midwives stayed. We have the rural health centre and we made a house so that MWs would stay, but they don’t stay… it took you all to reach here half a day… in the rainy season… is very tough”*

(Community member from hard to reach village in the FGD with community)

Community members indicated that they relied on the AMWs because they were local women and readily available for deliveries and in times of emergencies. Although AMWs were considered as reliable health care providers, some of the providers mentioned their concerns about over-confidence of the AMWs in conducting health care activities.

*“They [AMWs] think they can do all. We teach them ethics in school, meaning in the training but they act in the village as they were trained MW and say and act like a MW. I mean, we teach them and tell them what to follow ‘dos and don’ts’ during their training but they act and do beyond what is taught.”*

(Township level health care provider from key informant interview)

### Experiences of post-partum haemorrhage by AMWs

Although postpartum haemorrhage is a rare event in their daily practice, many of the AMWs in the study had experienced one or more serious events of a patient with severe bleeding, either in the community or at the hospital, during their service years. AMWs mentioned the events as unexpected and scary with serious ‘fear of death’ as their main concern due to limited experience and lack of available resources. The only measure AMWs were trained in and able to provide was timely referral to the nearest hospital and many felt helpless when they actually encountered the bleeding. The second level of care at rural health centres is not equipped with the lifesaving facilities for a bleeding woman in life-threatening danger Therefore, referral to a tertiary care hospital seemed to be the only resource for further care.

*“…I felt so scared as she was bleeding heavily and the MW told me to come to the hospital immediately and I put my fist into the vagina to stop the bleeding and I thought she was going to die”*

(AMW from hard-to-reach village during FGD with AMWs)

### Current practices and management of post-partum haemorrhage

Strong national guidelines exist for the government-trained skilled birth attendants at the community level (MWs) for the management of postpartum haemorrhage. Oxytocin injection is widely used in the facility settings, and is the intervention of choice. Management of postpartum haemorrhage at the community-level varied between MWs: most use 10 units of intramuscular oxytocin injection for prevention of postpartum haemorrhage, while a few MWs mentioned using misoprostol immediately after birth. The law prohibits AMWs from administering any injections, and current national guidelines do not support the use of misoprostol by AMWs. Misoprostol was introduced into the township health system around 2012. In the study Township, the drug became widely available with the introduction of the 3MDG program in 2014. One of the township level key informants described the availability of the drug as follows:

*“Nowadays, we have plenty of misoprostol and we mainly distribute to the MWs. For the AMWs, although it is in their manual, there is no clear guideline for distribution and usage by the central health authority… so what should we do”*

(Township level health care provider from key informant interview)

AMWs also seemed uncertain about how and where to procure it. An AMW from a rural area stated:

*“…we are willing to give misoprostol to the mothers, but currently only the MW give it, we are not allowed to give it…and we don’t have the drug either”*

(AMW in hard-to-reach area during FGD with AMWs)

According to the district level key informant interviews, each township seemed to have different practices with respect to misoprostol according to the township medical officer’s authority. In the study township, there seemed to be no restriction on the distribution of misoprostol to the AMWs by the MWs.

*“…the trend is changing [meaning involving all community base health workers] and we allow them [AMWs] to deliver non risk cases, you see old ones [AMWs] are delivering well and I think giving misoprostol would not be a bad idea. You see if our midwives are allowed to use misoprostol and they [AMWs] are not, it is hard for them to survive in the community. Our midwives give drugs and they [AMWs] are only allowed to rub the abdomen. I think that is not fair, they are the main persons in the community.”*

(Township level health care provider from key informant interview)

MWs in the study similarly mentioned that they were willing to give misoprostol to AMWs. Some of the MWs in the study had already distributed misoprostol to AMWs, especially in hard-to-reach areas and to those whom they trust.

*“We give the misoprostol together with the CDK [clean delivery kit] to all the AMWs in our areas. For those who are near to us, there is no problem because it is easy to monitor and refer if needed. But for those in the hill hard-to-reach, there is no-one apart from them. We tell them again and again when to give the drug [misoprostol], only after the birth of the baby and not to use it any other time”*

(MW assigned in the hard-to-reach area during FGD with MWs)

However, some of the key informant interviewees mentioned that they worried about the misuse of misoprostol to induce abortion, but MWs and AMWs in the FGDs did not mention anything in relation to abortion, potentially because induced abortions are illegal in Myanmar and are therefore a highly sensitive topic.

*“I always tell the AMWs to give the drug [misoprostol] only after the baby is born as it is very dangerous if is given before the delivery of the baby and because they are away from me during the time of the actual birth. I worry that they would use it early”*

(MW from hard-to-reach area during FGD with MWs)

One of the reasons for this concern was that the drug’s commonly used name in Myanmar language, “tha ein pwint say” (the ein: uterus; pwint: open; say: medicine), literally means “a drug that enhances the opening of the uterus”, creating misunderstanding among the users. Thus it was now purposely referred to as “tha ein kyunte say” (the ein: uterus; kyunte: contract; say: medicine), literally meaning contraction of the uterus, in most of the trainings.

*“You see they [AMWs] know it as “Tha ein pwint say” and we are worried that they would use it before delivery thinking that it will enhance labour. So we emphasize in the training as “tha ein kyunte say” and we stress that it can only be given after the birth of the baby*”

(Township level trainer of AMWs from key informant interview)

AMWs in the FGDs stated that they are willing and confident to take on the task of administering misoprostol during home deliveries. In addition, many of the AMWs expressed their frustration about not being allowed to provide any drugs to help a woman who is relying on her for care in time of danger.

*“When something happens, they [mothers] are with sad faces. They come to us with a small face [meaning a look of helplessness] saying we need you to treat us… The thing is that it is not that we cannot do it [give misoprostol], but we are not allowed to do it and we are scared to do it [scared of being punished rather than scared of giving the drug] …we are afraid”*

(AMW in hard-to-reach area during FGD with AMWs)

AMWs in the study considered that giving this life-saving drug would increase not only trust by the community but also increase their self-confidence in managing deliveries. However, some of the AMWs in the study highlighted the need for refresher training about postpartum haemorrhage and standardized dosage guidelines in using misoprostol.

*“we need training on postpartum haemorrhage and also how to prevent and treat before referring as is a life threatening event. We are confident to give the drug if we are trained properly and clearly teach us on how many tablets to give and when to give… we will follow what is taught”*

(AMW in hard-to-reach area during FGD with AMWs)

It became clear that there are no consistent guidelines at township level for distribution of misoprostol. The township level health care providers mentioned that they would be willing to distribute misoprostol to the AMWs if there was a clear written approval from the national level health authorities regarding the dosage and timing of drug delivery. Providers stressed that misoprostol should only be distributed to AMWs after systematic training about the benefits, side effects and correct time for usage.

### Training of AMW

According to the most recent AMW training curriculum in Myanmar, AMWs are taught a three-months theory and three-months practical training at the township or the respective station hospitals. Although delivery and postpartum care are meant to be taught through three hour long theoretical lectures each with 20 h of practical training, most of the participants in the study stated that theory was taught in a one-way lecturing format with minimal supervised practical training. Active management of the third stage of labour had been included in the training, however information provided for the use of misoprostol was inconsistent. Within the last five years, there have been three versions of the standard training guidelines in which the dose of misoprostol was different. The first guideline stated two tablets (400 μg), the second guideline did not specify the dosage and the latest guideline stated three tablets (600 μg) immediately after the birth of the baby. As a result, within the same township, AMWs had access to conflicting guidelines.

*“No, it [management of postpartum haemorrhage] was not taught… we were told to give [misoprostol] after the birth of the baby… not really clear explanation.”*

(AMW from non-hard-to-reach area during FGD with AMWs)

As a result, some of the AMWs explained that because the training was not clear, they had no option but to just refer patients to the health facility.

“*we don’t wait…we just refer as early as possible if is of risk…like multipara…*”

(AMW from non-hard-to-reach area during FGD with AMWs)

The information about the use of misoprostol as part of active management of third stage of labour by AMWs is included in the latest 2015 curriculum for AMWs, using a WHO standard dose of 600 μg (3 tablets) after the birth of the baby. However, the manual does not specifically state that AMW are allowed to administer misoprostol. Ongoing refresher trainings and newly trained AMWs in 2015 are said to be using the new guidelines.

## Discussion

Our work sought to reignite discussion on the potential for task shifting of misoprostol administration to AMWs by investigating the acceptability and feasibility of this intervention. The findings showed that AMWs generally were trained on the active management of third stage of labour, including the use of misoprostol, but not all AMWs reported using misoprostol in practice. Supportive reasons for task shifting administration of oral misoprostol to AMWs included discussions around the good relationship and trust between AMWs and MWs, whereby MWs felt confident distributing misoprostol to AMWs. However, the lack of clear government-level written permission to distribute the drug was perceived as a barrier to task shifting.

Overall in Myanmar, postpartum haemorrhage remains the leading cause of maternal mortality, accounting for 30% of maternal deaths. Equipping AMWs with the skills and resources to effectively prevent postpartum haemorrhage will be an essential component of efforts to reduce the incidence of postpartum haemorrhage. Although WHO guidelines recommend delivery of an injection of oxytocin as the first-line pharmacotherapy for the prevention and treatment of postpartum haemorrhage, the law in Myanmar restricts the use of injections by AMWs. Oral misoprostol is also an effective drug for the prevention of postpartum haemorrhage and is recommended by the WHO as a drug that could be used by AMWs in resource-limited settings [17]. Studies have also found that community-based distribution of misoprostol using a community level health worker was effective for prevention of postpartum haemorrhage [23–25]. According to a study done by Thein Thein Htay in 2007, the use of oral misoprostol by MWs at the community level has proven to be safe and effective for prevention of postpartum haemorrhage in Myanmar since 2007 and was considered for nationwide distribution to MWs and AMWs [26]. However, competing priorities such as disease control activities over the past decade and frequent changes in policy-makers have hampered progress towards this goal.

A major enabling factor to task shifting misoprostol administration to AMWs was the recognition amongst community members, healthcare providers and policy makers of the critical role played by AMWs in providing care during childbirth, particularly in hard-to-reach areas. Home births remain common in Myanmar with over 70% of deliveries occurring outside of a health facility [27]. Our findings suggest this to be due to a combination of factors including convenience, cultural preference and significant geographical constraints, which restrict the accessibility of healthcare facilities by people in the hard to reach areas. Thus the roles of MWs and AMWs are essential as these community-based providers are often called upon to attend home births. Studies have also shown that community health workers are competent not only in pregnancy and childbirth practices, but also in providing other health related services such as nutrition promotion, immunization and disease control activities [28, 29].

In our study area, AMWs were considered by community members and key informants to be capable providers of care during childbirth. As such, distribution of oral misoprostol to AMWs was considered by the midwives and key informants sampled in this study to be a potentially effective and feasible option for the prevention of postpartum haemorrhage where skilled birth attendants are not available. AMWs themselves reported to be able and willing to take on the task of misoprostol administration and welcomed the opportunity to expand their capacity to provide improved care during delivery.

A number of barriers to task shifting of misoprostol to AMWs were identified by study participants. Some key informants mentioned fear of untimely misoprostol use before the child is born, or when used to induce abortion, particularly if sufficient training and standardised policies and procedures were not in place. Interestingly, the use of misoprostol to induce abortion was not mentioned by MWs and AMWs, likely because they know it is illegal to use misoprostol for this indication. Studies on the use of misoprostol for prevention of postpartum haemorrhage have helped to address these concerns, and a comprehensive review of 18 programs using lay health care workers to provide misoprostol found very low rates of incorrect use [23–25]. This suggests that AMWs in Myanmar have considerable potential to administer misoprostol correctly if provided with sufficient training. AMWs themselves also spoke about inconsistent quality of training, and some highlighted a paucity of information in their training regarding postpartum haemorrhage and the rationale for using misoprostol. A review done by Prata et al. in 2013 stated that along with a national policy and drug distribution mechanisms for misoprostol, adequate provision of information and training should be included for lay health workers and the community [23]. The findings suggest that MWs and township-level health providers would be confident in the ability of AMWs to administer misoprostol correctly with additional refresher trainings. Some of the AMWs in our study were already administering misoprostol, further highlighting the need for clear guidelines, consistent policies and adequate training.

This study suggests that oral misoprostol is a viable option to prevent postpartum haemorrhage especially for hard-to-reach areas where AMWs are the primary attendants for home births.

### Limitations of the study

The study township was purposively selected based on its ‘hard-to-reach’ characteristics and as such, findings might not be representative of the perspectives of equivalent stakeholders in other townships in Myanmar, potentially limiting generalization of the findings to other areas. The relationships between the AMWs and the MWs in the study were very positive and it may be due to the social desirability bias of their relationship that actual practice may vary.

## Conclusion

With over 30% of the maternal deaths in Myanmar due to postpartum haemorrhage, it is time that health planners consider this evidence-based intervention for scale-up throughout the country. The findings of this study support the feasibility of task shifting of misoprostol to AMWs as a crucial intervention for the prevention of postpartum haemorrhage in Myanmar.

## Additional file

Additional file 1: Interview guides for focus group discussions and in-depth interviews, used as part of the study are available in the supplementary files, and entitled:” Qualitative interview guides and questionnaire”. (DOCX 54 kb)

## Abbreviations

3MDG: The Three Millennium Development Goal Fund
AMW: Auxiliary midwife
COREQ: The consolidated criteria for reporting qualitative research
FGD: Focus group discussion
IDI: In-depth interviews
MW: Midwife
WHO: World Health Organization
