# Supplementary material for: Prevention of postpartum haemorrhage by community-based auxiliary midwives in hard-to-reach areas of Myanmar: a qualitative inquiry into acceptability and feasibility of task shifting
Source: BMC Pregnancy Childbirth. 2017 May 17;17:146. doi: 10.1186/s12884-017-1324-6 (PMC5436430; doi:10.1186/s12884-017-1324-6)
Supplement: Additional file 1: — Interview guides for focus group discussions and in-depth interviews, used as part of the study are available in the supplementary files, and entitled:” Qualitative interview guides and questionnaire”. (DOCX 54 kb) [file 12884_2017_1324_MOESM1_ESM.docx]

## Focus group discussion guides and key informant interview guides for Midwives, Auxiliary Midwives, Mothers, Community members, Policy makers and Health Care Providers used for the study entitled:”The role of Auxiliary Midwives in community-based maternal and child health care in Myanmar: an assessment of the feasibility of task shifting”

Notes:

Brief questionnaires were used before the focus group discussions and key informant interviews to obtain participant characteristics.

Guides and questionnaires are presented in standard English language. All questionnaires were translated into Myanmar language for use in the study.

## Participant group: Mothers with under three year old children

## Focus Group Discussion Guide and characteristics questionnaire (standard English-language version)

We invite you to answer the following questions that will tell us a little more about you and your background. This questionnaire is part of the study on role of AMWs in your community being done by Department of Health and Burnet Institute. You can choose not to answer any question. Your privacy will be respected and you do not have to provide your name. When the study results are published or shared, no names or identifying information will be used.

| Age (in completed years) |  |
| --- | --- |
| Marital Status   1. Single 2. Married 3. Divorced/ Separated 4. Widowed |  |
| What is the highest level of education attained?   1. Primary 2. Secondary 3. High School 4. University 5. Graduate |  |
| Number of children currently alive? |  |
| Age of the youngest child (in months) |  |
| Place of Childbirth of the last child?   1. Home 2. Facility (Government /Private) 3. Others (specify)_____________ |  |
| Who was the main person assisting your delivery?   1. Doctor 2. LHV/HA 3. MW 4. AMW 5. TBA 6. Others (specify)_________________ |  |
| Did you use any medication during/after your last childbirth?  If so please specify :   1. Oral drugs (Western) Yes (1) No (0) 2. Traditional herbal Medicine (Oral) Yes (1) No (0) 3. Traditional herbal Medicine (Inhalant) Yes (1) No (0) 4. Injections Yes (1) No (0)   Others (specify) |  |
| Did you have any complications with your last delivery?  If yes please specify: |  |
| What is your average monthly family income? (in Kyats) |  |

**Themes, Questions and Probes**

**Current Practices around childbirth**

Can you all tell me about how women in your community practice childbirth?

- - *With whom do women usually give childbirth?*
  - *Where do women give childbirth normally?*
  - *What are the reasons for giving childbirth at home or at facility?*
  - *Who makes the decision around childbirth?*

**Medications at the time of childbirth**

Can you tell me about medications that are used during or after childbirth?

- *Do women use any medications during or after childbirth?*
- *Would women want medication used at childbirth? Why/why not?*
- *What types of medication are used?*
- *Who normally provides medication around the time of childbirth?*
- *How much do they cost (if known)?*
- *Who decides what medications are used?*

**Types of health care providers in the village and their role**

Can you tell me how many health care providers are in the village for women and children?

- - *Who they are?*
  - *How are they different from each other in term of age and experience?*
  - *How are they different in terms of ability to access services? Probe: Why?*
  - *How are they different from each other in terms of quality of care?* *Probe: Why?*
  - *How are they different in providing antenatal care, childbirth and post-natal care? Probe: Why?*
  - *How are they different in providing other services (immunization, contraception etc.)?* *Probe why?*
  - *How do they differ in other roles? (community activities, counseling and giving advice)?Probe why?*

How do families in the village choose providers for care during childbirth?

- - *Social relationship, trust, cost, availability etc.*
- *How was the decision made?*
- *What are the most important factors in making this decision?(money, trust, availability)*
- *Who is involved in the decision making?*

**Attitudes towards AMWs**

What do women in your village think about the AMWs in your village?

- *What is the impression on her skill?*
- *Does she meet the expectations? Why/ why not (satisfied/not)*
- *What do you value most about her?*
- *Do you expect her to do more things and can you tell me what they are?*
- *What are the benefits of using an AMW?*
- *What are the reasons for NOT using an AMW?*

What are the barriers to women using an AMW in your village?

- *How is her accessibility?*
- *How is her availability?*
- *Does she charge for her service and how much?*
- *Do people in the community like and trust her? Probe: why/why not*

**Expectation of the services provided by AMWs**

What services do you expect AMWs to be able to provide?

- *Preventive care services (health education, counseling etc)*
- *Curative care services (e.g. giving medication and injections)*
- *Is there anything you would expect her to do that she is not currently doing?*

Is the community happy to have an AMW?

- *Is there a benefit of having an AMW in your village (why/why not)*
- *What changes could you see after having an AMW in your village?*

**Task shifting possibilities**

What would/do the community think about AMWs giving oral medications to women during or after childbirth?

- *Is it acceptable or not acceptable?*
- *Do you think it is/would be effective?*

What would the community think about AMWs trying to resuscitate newborn babies that are not breathing?

- *Is it acceptable or not acceptable?*
- *Do you think it is/would be effective?*

Is there anything you all would like to add or give suggestions for AMWs in your village or in other villages?

## Participant group: Community members

## Focus Group Discussion Guide and characteristics questionnaire (standard English-language version)

We invite you to answer the following questions that will tell us a little more about you and your background. This questionnaire is part of the study on role of AMWs in your community being done by Department of Health and Burnet Institute. You can choose not to answer any question. Your privacy will be respected and you do not have to provide your name. When the study results are published or shared, no names or identifying information will be used.

| Age (in completed years) |  |
| --- | --- |
| Sex Male (1) Female (2) |  |
| Marital status   1. Single (Skip question on number of child) 2. Married 3. Divorced/ Separated 4. Widowed |  |
| What is the highest level of education attained?   1. Primary 2. Secondary 3. High School 4. University 5. Graduate |  |
| Occupation   1. Dependent/ retired 2. Manual Laborer 3. Farmer/ Crop production 4. Own business (Small) 5. Own business (Large) 6. Government Employee 7. Others (specify) |  |
| Number of children currently alive? |  |
| What is your average monthly income? (in Kyats) |  |

**Themes, Questions and Probes**

**Current Practices around childbirth**

Can you all tell me about how women in your community practice delivery?

- - *With whom do women usually give childbirth?*
  - *Where do women give childbirth normally?*
  - *What are the reasons for giving childbirth at home or at facility?*
  - *Who makes the decision around childbirth?*

**Medications at the time of childbirth**

Can you tell me about medications that are used during or after childbirth?

- *Do women use any medications during or after childbirth?*
- *Would women want medication used at childbirth? Why/why not?*
- *What types of medication are used?*
- *Who normally provides medication around the time of childbirth?*
- *How much do they cost (if known)?*
- *Who decides what medications are used?*

**Types of health care providers in the village and their role**

Can you tell me how many health care providers are in the village for women and children?

- - *Who they are?*
  - *How are they different from each other in term of age and experience?*
  - *How are they different in terms of ability to access services? Probe:why?*
  - *How are they different from each other in terms of quality of care? Probe: why?*
  - *How are they different in providing antenatal care, childbirth and post-natal care? Probe:why?*
  - *How are they different in providing other services (immunization, contraception etc.)? Probe:why?*
  - *How do they differ in other roles? (community activities, counseling and giving advice)?* *Probe:why?*

How do families in the village choose providers for care during childbirth?

- - *Social relationship, trust, cost, availability etc.*
- *How was the decision made?*
- *What are the most important factors in making this decision?*
- *Who is involved in the decision making?*

**Attitudes towards AMWs**

What do people in your village think about the AMW in your village?

- *What is your impression on her skill?*
- *Does she meet your expectations? Why/ why not (were u satisfied)*
- *What do you value most about her?*
- *Do you expect her to do more things and can you tell me what they are?*
- *What is the benefit of using an AMW?*
- *What are the reasons for NOT using an AMW?*

What are the barriers to women using AMW in your village?

- *How is her accessibility?*
- *How is her availability?*
- *Does she charge for her service and how much?*
- *Do people in the community like and trust her?Probe: why/ why not*

**Expectation of the services provided by AMWs**

What services do you expect AMWs to be able to provide?

- *Preventive care services (health education, counseling etc)*
- *Curative care services (e.g. giving medication and injections)*
- *Is there anything you would expect her to do that she is not currently doing?*
- *Is there a benefit of having a AMW in your village (why/why not)*
- *What changes could you see after having an auxiliary midwife in your*

*community ?*

**Task shifting possibilities**

What would/do the community think about AMWs giving oral medications to women during or after childbirth?

- *Is it acceptable or not acceptable?*
- *Do you think it is/would be effective?*

What would the community think about AMWs trying to resuscitate newborn babies that are not breathing?

- *Is it acceptable or not acceptable?*
- *Do you think it is/would be effective?*

Is there anything you all would like to add or give suggestions for AMWs in your village or in other villages?

## Participant group: Auxiliary Midwives (AMWs)

## Focus Group Discussion Guide and characteristics questionnaire (standard English-language version)

We invite you to answer the following questions that will tell us a little more about you and your background. This questionnaire is part of the study on role of AMWs in your community being done by Department of Health and Burnet Institute. You can choose not to answer any question. Your privacy will be respected and you do not have to provide your name. When the study results are published or shared, no names or identifying information will be used.

| Age (in completed years) |  |
| --- | --- |
| Marital status   1. Single 2. Married 3. Divorced/ Separated 4. Widowed |  |
| What is the highest level of education attained?   1. Primary 2. Secondary 3. High School 4. University 5. Graduate |  |
| Years of practice as an AMW (in years) |  |
| Average number of deliveries per year as a primary birth attendant (in numbers) |  |
| When were you trained as an AMW? (exact year) |  |
| How long was this training (in weeks/ months/ years) |  |
| When was the last training that you received on maternal and child health? (exact year) |  |
| Occupation   1. Dependent/ retired 2. Manual Laborer/ Daily wager 3. Farmer/ Crop production 4. Own business (Small) 5. Own business (Large) 6. Government Employee 7. Only works as an AMW 8. Others (specify) |  |
| Do you give any medication during/after childbirth to women?  If so please specify:   1. Oral drugs (Western) Yes (1) No (0) 2. Traditional herbal Medicine (Oral) Yes (1) No (0) 3. Traditional herbal Medicine (Inhalant) Yes (1) No (0) 4. Injections Yes (1) No (0) 5. Others (specify) |  |

**Themes, Questions and Probes**

**Current Practices around childbirth**

Can you tell me what you do during childbirth for women in your community?

- - *Can you tell me how you take care of a woman during childbirth*
- *Do you manage it alone or were you helped by others? (family members, TBAs etc.)?*
- *Have you encountered any emergencies?*
- *How was it managed?*
- *How do you refer a woman? (When, how?)*

**Practice of medications at the time of childbirth**

Can you tell me about medications that are used during or after childbirth?

- *Do you use/practice any type of medicines for childbirth and post-natal*

*periods? (oral, injections, traditional medicine)*

- *Do women in the community demand/ expect for any type of medication*

*during and after childbirth? Why/why not?*

- *What types of medication are used?*
- *How did you get the medicine?*
- *How about the cost?*

**Types of health care providers in the village and their role**

Can you all tell me how many health care providers are in the village for women and children?

- *Who they are? (MWs, TBAs and others)*
- *How are they different from you in term of skill, age and experience?*
- *In your opinion are the roles different from each other? (Role difference and overlap)*
- *Do you collaborate with them? If so, how?*
- *Joint practices and helping each other?*
- *Have you ever experienced difficulties in dealing with MWs, TBAs or other healthcare providers?What type of women chooses to deliver with you and why?*

**Challenges of providing services as AMWs**

Can you tell me the difficulties in carrying out your role as an AMW?

- *What are the major challenges of being an AMW?*
- *What is needed to fulfil your role?*
- *Do you think you have enough skill to carrying out your assigned activities?*
- *Do you think that you were trained well during your training?*
- *Do you have all the necessary equipment and supplies to carry out your activities?*

What are the challenges faced in providing quality care during the time of childbirth?

- *What do they expect you to do?*
- *What are the main barriers in fulfilling the community expectations?*
- *Was the community supportive of your activities (Yes/no, WHY)*
- *Do you have any difficulties dealing with the community?*

Do you think you need more support?

- *In what ways? (incentives, moral, financial support, supervision)*
- *From who? (Community, health care provider: midwives, TMO etc.)*
- *What? (skill, equipment, training etc)*

**Feasibility of the four specific tasks for AMWs**

As an AMW, do you think providing the following interventions would be feasible and acceptable?

- *Have you given any oral medication to pregnant women?*
- *If you need to be giving oral medication what are the challenges?*
- *Have you ever used misoprostol to prevent PPH?*
- *Do you think it will be feasible to provider oral misoprostol to prevent PPH? What are the challenges?*
- *Have you ever used misoprostol to treat PPH?*
- *Do you think it will be feasible to provide oral misoprostol to treat PPH? What are the challenges?*
- *Have you ever used oral antibiotics to treat puerperal sepsis?*
- *Do you think it will be feasible to provide oral antibiotics to treat puerperal sepsis? What are the challenges?*
- *Have you ever resuscitate a newborn using bag and mask?*
- *Do you think it will be feasible to resuscitate a newborn using bag and mask? What are the challenges?*

## Participant group: Midwives (MWs)

## Focus Group Discussion Guide and characteristics questionnaire (standard English-language version)

We invite you to answer the following questions that will tell us a little more about you and your background. This questionnaire is part of the study on role of AMWs in your community being done by Department of Health and Burnet Institute. You can choose not to answer any question. Your privacy will be respected and you do not have to provide your name. When the study results are published or shared, no names or identifying information will be used.

| Age (in completed years) |  |
| --- | --- |
| Marital status  1. Single  2. Married  3. Divorced/ Separated  4. Widowed |  |
| What is the highest level of educational attained (specify)   1. Primary 2. Secondary 3. High School 4. University 5. Graduate |  |
| When did you start working as a MW? (exact year) |  |
| How long have you been posted in this township? (in years) |  |
| Average number of deliveries per year as a primary birth attendant? (in numbers) |  |
| How long have you been supervising AMWs (in years) |  |
| Do you give any medication during/after childbirth to women?  If so please specify:   1. Oral drugs (Western) Yes (1) No (0) 2. Traditional herbal Medicine (Oral) Yes (1) No (0) 3. Traditional herbal Medicine (Inhalant) Yes (1) No (0) 4. Injections Yes (1) No (0) 5. Others (specify) |  |

**Themes, Questions and Probes**

**Current Practices around childbirth**

Can you tell me how you manage childbirth and post-partum period for women in your community?

- *What do you normally do when a women seeks you for childbirth and postnatal care?*
- *Do you manage it alone or were you helped by others?(family members, TBAs, AMWs etc)*
- *Have you encountered any emergencies?*
- *How was it managed?*
- *How do you refer a woman? (when, how?)*

**Practice of medications at the time of childbirth**

Can you tell me about medications that are used during or after childbirth?

- *Do you use/practice any type of medicines for child childbirth and post-natal*

*periods? (oral, injections, traditional medicine)*

- *Do women in the community demand/ expect for any type of medication*

*during and after childbirth? Why/why not?*

- *What types of medication are used?*
- *How did you get the medicine?*
- *How about the cost?*

**Types of health care providers in the village and their role**

Can you all tell me how many health care providers are in the village for women and children?

- *Who they are? (AMWs, TBAs and others)*
- *How are they different from you in term of skill, age and experience?*
- *In your opinion are the roles different from each other? (Role overlap)*
- *Do you collaborate with them? If so, how?*
- *Joint practices and helping each other?*
- *Have you ever experienced difficulties in dealing with AMWs, TBAs or other healthcare providers?*
- *What type of women chooses to deliver with you and why?*

**Attitude towards AMWs**

Can you tell me what you all think about AMWs?

- *What is your impression on her skill and her performance?*
- *What is your expectation towards AMWs?*
- *Does she meet your expectations? Why/ why not (satisfied/not)*
- *Do you expect her to do more things and can you tell me what they are?*
- *What do you value most about her?*
- *Is there anything you would expect her to do that she is not currently doing?*
- *Is there a benefit of having a AMW (why/why not)*
- *What changes could you see after having an AMWs in your*

*community?*

**Feasibility of the four specific tasks for AMWs**

As an MW do you think it is feasible and acceptable for AMWs to provide the following interventions?

- *Oral medication to pregnant women?*
- *Oral misoprostol to prevent PPH? What are the challenges?*
- *Oral misoprostol to treat PPH? What are the challenges?*
- *Oral antibiotics to treat puerperal sepsis? What are the challenges?*
- *Resuscitating a newborn using bag and mask?*
- *In what circumstances do you think it would be ok for AMWs to provide these services (places, training, situations etc)? What are the challenges?*

## Participant group: Policymakers and Health Care Providers

## Key Informant Interview Guide and characteristics questionnaire (standard English-language version)

We invite you to answer the following questions that will tell us a little more about you and your background. This questionnaire is part of the study on role of AMWs in your community being done by Department of Health and Burnet Institute. You can choose not to answer any question. Your privacy will be respected and you do not have to provide your name. When the study results are published or shared, no names or identifying information will be used.

| Age (in completed years) |  |
| --- | --- |
| Highest level of educational attainment (specify)   1. Primary 2. Secondary 3. High School 4. University 5. Graduate |  |
| How long have you worked in the government service?(in years) |  |
| What is your current post? (specify) |  |
| How long have you been posted in the current position? (in years) |  |
| What is your role in AMW program at your township/position (specify)   1. Recruitment Yes (1) No (0) 2. Training Yes (1) No (0) 3. Supervision Yes (1) No (0) 4. Planning Yes (1) No (0) 5. Others (specify) |  |

**Themes, Questions and Probes**

**Types of health care providers in the village and their role**

Can you tell me how many health care providers are at the community level for women and children?

- *Who they are?*
- *How are they different from each other in term of skill, age and experience?*
- *How are they different in providing antenatal care, childbirth and post-natal care?*
- *How are they different in providing other services (immunization, contraception etc.)?*
- *How do they differ in other roles? (community activities, counseling and giving advice)?*

Can you tell me the current role of AMW in maternal and child health care?

Do you think AMW are performing beyond her role?

- *Hard to reach areas*
- *Unreached by supervision*
- *What types of activities are carried out?*
- *What is your opinion?*

**Attitude towards AMW**

What is your opinion about the need for AMW and whether they are fulfilling that need?

- *Necessary cadres or not?*
- *What do you think of their skills?*
- *What do you think of their performance with in the community?*
- *Any concerns towards their role?*

**Effectiveness of the AMW programme**

What is the main aim of the AMW programme?

Do you think the objectives of the programme have been met or will be met in the future? Why/why not?

What are the main challenges of the AMW programme in your township?

- *Recruitment*
- *Training*
- *Supervision*
- *Logistics*
- *Reports and feedback*

How are these difficulties being managed?

**Task shifting possibilities**

Have you ever heard of the task shifting guideline before?

What tasks do you think AMW can perform?

What will be the pros and cons of adding new task to the AMWs role?

Do you think it is feasible and acceptable for AMWs to provide the following interventions?

- Oral medication to pregnant women?
- Oral misoprostol to prevent PPH? What are the challenges?
- Oral misoprostol to treat PPH? What are the challenges?
- Oral antibiotics to treat puerperal sepsis? What are the challenges?
- Resuscitating a newborn using bag and mask?

In what circumstances do you think it would be ok for AMWs to provide these services (places, training, situations etc)?

What are the challenges?

What are the necessary skills, equipment and procedures?
